# Neonatal and child health crises due to recent floods in Pakistan

**DOI:** 10.1016/j.amsu.2022.104837

**Published:** 2022-11-09

**Authors:** Sidhant Ochani, Syeda Ilsa Aaqil, Abubakar Nazir, Fatima Binte Athar, Kaleem Ullah

**Affiliations:** Department of Medicine, Khairpur Medical College, Khairpur Mir's, Pakistan; Department of Medicine, Jinnah Sindh Medical University, Karachi, Pakistan; Department of Medicine, King Edward Medical University, Lahore, Pakistan; Department of Medicine, Karachi Medical and Dental College, Karachi, Pakistan; Department of Liver Transplantation, Pir Abdul Qadir Shah Institute of Medical Sciences, Gambat, Pakistan

## Abstract

Neonates and children are more vulnerable to the negative impact of flood-related changes and may have a variety of detrimental negative impacts on their health. They are more prone to get various infectious diseases. They are also more vulnerable to malnutrition during floods. Flooding limits access to clean water as sewage overflows and contaminates nearby water sources. The polluted setting in the flood-affected area makes it difficult to ensure the hygiene of feeding equipment used to prepare infant formula. Breastfeeding may also become less effective due to the lack of privacy for women to breastfeed their kids while living in temporary shelters with other flood victims. In addition, milk production decreases and might even cease due to mothers’ reduced food intake and increased stress levels. Flooding may also cause supplemental feeding to deteriorate. The mothers and other primary caregivers usually lack the resources in affected areas to prepare supplemental diets for their kids, which further harm the babies. There is mounting evidence that children are more likely to develop clogged noses, itchy eyes, hoarseness, skin complications, and sneezing while living in humid areas.

Flooding is the most frequent and devastating natural calamity worldwide, with increased morbidity and mortality. Over the past three decades, flood catastropheshave harmed over 2.8 billion people globally and have caused the death of over 200,000 people. The effect of floods on a population's health varies depending on the population's vulnerability. It greatly impairs the quality of life of everyone in the affected area. However, neonates and children are more vulnerable to the negative impact of flood-related changes and may have a variety of detrimental negative impacts on their health [1].

Infants and children are more prone to get various infectious diseases. They are also more vulnerable to malnutrition during floods. Flooding limits access to clean water as sewage overflows and contaminates nearby water sources. The polluted setting in the flood-affected area makes it difficult to ensure the hygiene of feeding equipment used to prepare infant formula [2]. Breastfeeding may also become less effective due to the lack of privacy for women to breastfeed their kids while living in temporary shelters with other flood victims. In addition, milk production decreases and might even cease due to mothers’ reduced food intake and increased stress levels. Flooding may also cause supplemental feeding to deteriorate. The mothers and other primary caregivers usually lack the resources in affected areas to prepare supplemental diets for their kids, which further harm the babies [3]. Children consume more water per unit of body mass than adults, and that is why they are particularly vulnerable to severe water-borne microbial morbidity and mortality [4]. There is mounting evidence that children are more likely to develop clogged noses, itchy eyes, hoarseness, skin complications, and sneezing while living in humid areas [5].

Pakistan is ranked 14th out of 163 nations and regions in the Children's Climate Risk Index (CCRI). The terrible storms, floods, and landslides during high monsoon rains this year in Pakistan have affected more than thirty-three million people, including over sixteen million children. Approximately 1,100 people, including more than 350 children, have died, and more than 1,600 have been injured. Houses, and vital infrastructures, such as roads, bridges, schools, hospitals, and public health facilities, have also been destroyed [6]. Malaria, dengue, and acute diarrhea outbreaks have been reported frequently after recent flooding. According to reports, major damage to the educational infrastructure has been done, and approximately 17,566 schools have been reported to be damaged or destroyed, further compromising children's education. There have already been reports of other water-borne illnesses, respiratory infections, and skin conditions in children [7].

Humanitarian aid is required for around three million children. Children and nursing mothers should have free access to purified water. Chlorination and iodination of tap water should be done frequently for disinfection and to kill disease-causing organisms. These steps must be ensured to prevent water-borne diseases like typhoid, malaria, and dengue. Surveillance should be conducted for timely identification of disease outbreaks, nutritional status, and shortage of food resources. A properly balanced diet supply must be ensured to prevent growth retardation in children. Polio vaccines in high-risk flood areas should be administered as there is an increased risk of poliovirus transmission due tothe consumption of contaminated water. Mass vaccination for children against measles and cholera, hepatitis A and Earealso extremely important. Also, skilled medical professionals should be available in flood areas to care for newly born babies.

## Ethical approval

Not Applicable.

## Sources of funding

The author(s) received no financial support for the research, authorship, and/or publication of this article.

## Author contribution

Literature Review was done by all authors. Manuscript was written by **SO**, **SIA**, **AN** and **FBA**. Review editing, formatting and referencing was done by **SO** and **KU**.

## Consent

Not Applicable.

## Registration of research studies

Not Applicable.

## Guarantor

All authors accept full responsibility for the work and/or the conduct of the study, had access to the data, and controlled the decision to publish.

## Declaration of competing interest

The author(s) declared no potential conflicts of interest concerning the research, authorship, and/or publication of this article.
